# Atmospheric pathways of chlorinated pesticides and natural bromoanisoles in the northern Baltic Sea and its catchment

**DOI:** 10.1007/s13280-015-0666-4

**Published:** 2015-05-28

**Authors:** Terry Bidleman, Kathleen Agosta, Agneta Andersson, Eva Brorström-Lundén, Peter Haglund, Katarina Hansson, Hjalmar Laudon, Seth Newton, Olle Nygren, Matyas Ripszam, Mats Tysklind, Karin Wiberg

**Affiliations:** Department of Chemistry, Umeå University, Linnaeus väg 6, 901 87 Umeå, Sweden; Department of Ecology and Environmental Science, Umeå University, Linnaeus väg 6, 901 87 Umeå, Sweden; IVL Swedish Environmental Research Institute, P.O. Box 530 21, 40014 Göteborg, Sweden; Swedish University of Agricultural Sciences (SLU), 901 83 Umeå, Sweden; Department of Environmental Science and Analytical Chemistry (ACES), Stockholm University, Svante Arrhenius väg 8, 106 91 Stockholm, Sweden; Department of Aquatic Sciences and Assessment, Swedish University of Agricultural Sciences (SLU), P.O. Box 7050, 750 07 Uppsala, Sweden

**Keywords:** Baltic Sea, Bothnian Bay, Chlorinated pesticides, Natural brominated compounds, Atmospheric deposition, Air–sea exchange

## Abstract

Long-range atmospheric transport is a major pathway for delivering persistent organic pollutants to the oceans. Atmospheric deposition and volatilization of chlorinated pesticides and algae-produced bromoanisoles (BAs) were estimated for Bothnian Bay, northern Baltic Sea, based on air and water concentrations measured in 2011–2012. Pesticide fluxes were estimated using monthly air and water temperatures and assuming 4 months ice cover when no exchange occurs. Fluxes were predicted to increase by about 50 % under a 2069–2099 prediction scenario of higher temperatures and no ice. Total atmospheric loadings to Bothnian Bay and its catchment were derived from air–sea gas exchange and “bulk” (precipitation + dry particle) deposition, resulting in net gains of 53 and 46 kg year^−1^ for endosulfans and hexachlorocyclohexanes, respectively, and net loss of 10 kg year^−1^ for chlordanes. Volatilization of BAs releases bromine to the atmosphere and may limit their residence time in Bothnian Bay. This initial study provides baseline information for future investigations of climate change on biogeochemical cycles in the northern Baltic Sea and its catchment.

**Electronic supplementary material:**

The online version of this article (doi:10.1007/s13280-015-0666-4) contains supplementary material, which is available to authorized users.

## Introduction

**Fig. 1:**
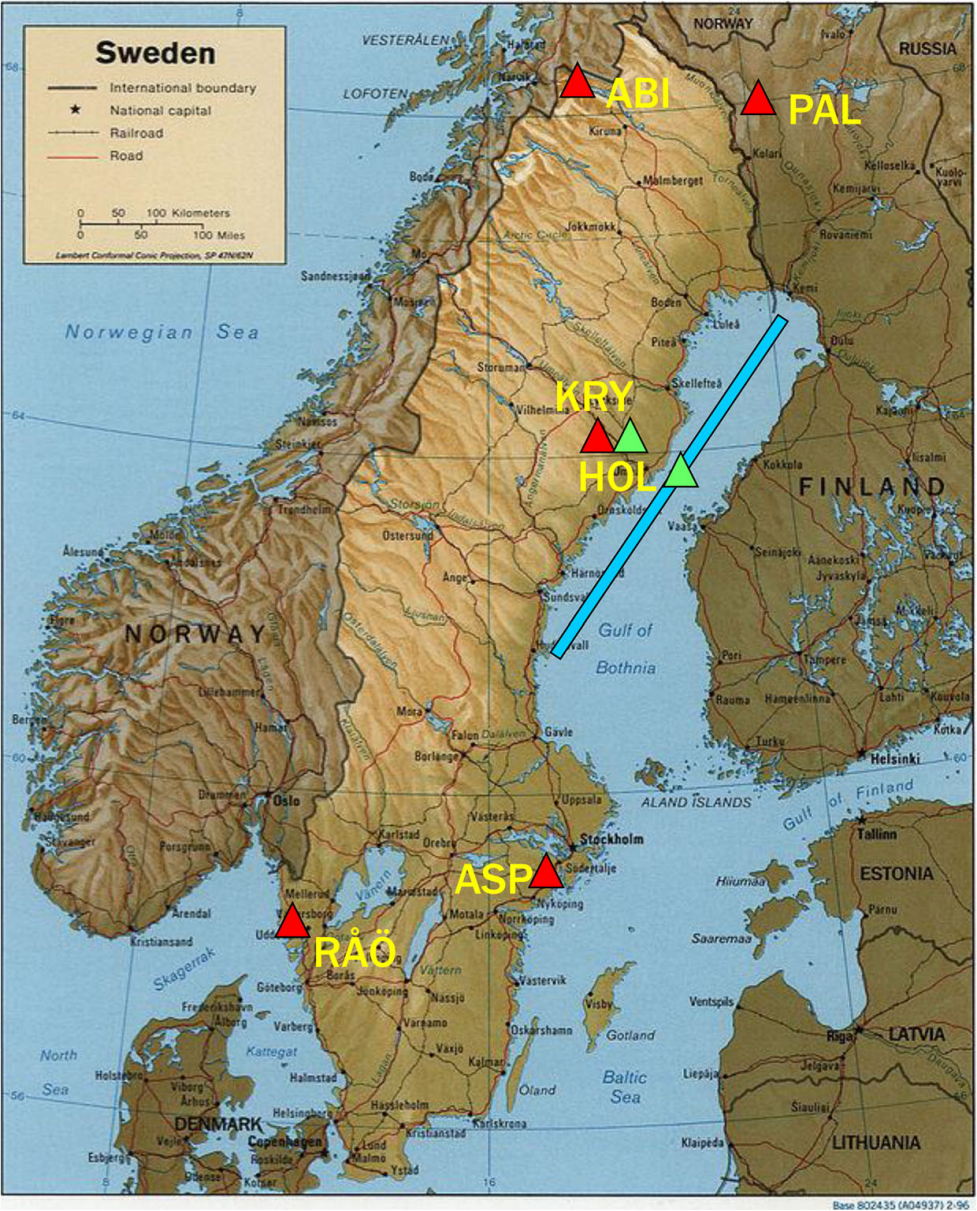
Deposition stations at Abisko (ABI), Pallas (PAL), Krycklan (KRY), Aspvreten (ASP), and Råö (RÅÖ) (*red triangles*) and air stations at KRY and Holmön (HOL) (*green triangles*). *Blue bar* shows range of water sampling locations in Bothnian Bay–Bothnian Sea

The Baltic Sea is impacted by synthetic organic chemicals released from various sources, such as use in industry and consumer products, generated by incomplete combustion, and applied as pesticides (HELCOM 2009, 2010). The mixture is further complicated by natural brominated compounds that are synthesized by macroalgae and phytoplankton. Long-range atmospheric transport and deposition are large, and in many cases the dominant, processes for delivering persistent organic pollutants (POPs) to the oceans (Lohmann et al. 2007). This paper reports sources and pathways of halogenated compounds in the Gulf of Bothnia, which is divided into upper Bothnian Bay and lower Bothnian Sea (Fig. 1). The main aim is to compare atmospheric pathways for chlorinated pesticides, which arrive by atmospheric transport and deposit to the sea surface and catchment, and natural bromoanisoles (BAs) that are generated within the Baltic. The potential influence of climate change on their sources and pathways is discussed.

### Chlorinated pesticides

The pesticides dichlorodiphenyltrichloroethane (DDT), chlordane (CHL), heptachlor (HEPT), hexachlorobenzene (HCB), and dieldrin (DIEL) were among the original “dirty dozen” POPs banned by the Stockholm Convention in 2001, and hexachlorocyclohexanes (HCHs) were added in 2009 (UNEP 2014) (all acronyms and compound abbreviations are explained in Box 1). Former use of HCB as a fungicide was estimated to dominate emissions in the 1950s and 1960s. HCB was also used in industry and continues to be released as a by-product of chlorinated solvent and pesticide manufacturing, incomplete combustion, and from old dumpsites. Estimated current emissions are 70–95 % lower than those in the 1970s (Barber et al. 2005). Pesticides volatilize from areas of former use and by “secondary” emissions of residues in background soil, water, and vegetation (Cabrerizo et al. 2011; Bidleman et al. 2012). Long-term monitoring shows general worldwide declines in air concentrations (Kong et al. 2014). Stabilizing or slightly increasing levels of some compounds after about 2000 (Becker et al. 2012; Hung et al. 2013) have been correlated with loss of sea ice cover and increased surface air temperature (Ma et al. 2011).

**Box 1:** Acronyms and compound abbreviations in alphabetical order

BA	Bromoanisole
BP	Bromophenol
CC	*Cis*-chlordane
CHL	Chlordane
CI	Confidence interval
CN	*Cis*-nonachlor
CPF	Chlorpyrifos
CUP	Currently used pesticide
DAC	Dacthal
DDE	Dichlorodiphenyldichloroethene
DDT	Dichlorodiphenyltrichloroethane
DBA	Dibromoanisole
DIEL	Dieldrin
DOC	Dissolved organic carbon
EMEP	European Monitoring and Evaluation Program
ENDO	Endosulfan
ENSUL	Endosulfan sulfate
GFF	Glass fiber filter
HCB	Hexachlorobenzene
HCH	Hexachlorocyclohexane
HELCOM	Helsinki Commission
HEPT	Heptachlor
HEPX	Heptachlor *exo*-epoxide
HNP	Halogenated natural product
LOD	Limit of detection
MeO-BDE	Methoxylated polybrominated diphenyl ether
OH-BDE	Hydroxylated polybrominated diphenyl ether
PBDD	Polybrominated dibenzo-*p*-dioxin
PBDE	Polybrominated diphenyl ether
PCDD	Polychlorinated dibenzo-*p*-dioxin
PCDF	Polychlorinated dibenzofuran
PCB	Polychlorinated biphenyl
POC	Particulate organic carbon
POP	Persistent organic pollutant
PUF	Polyurethane foam
RSD	Relative standard deviation
TBA	Tribromoanisole
TBP	Tribromophenol
TC	*Trans*-chlordane
TEQ	Toxic equivalents (dioxin type)
TN	*Trans*-nonachlor
UNEP	United Nations Environment Program
XAD	Polystyrene-divinyl benzene copolymer

The insecticide endosulfan (ENDO) is among the “substances of specific concern to the Baltic Sea” (HELCOM 2009). Technical-grade ENDO comprises two isomers, ENDO-I and ENDO-II, in ratios from 2:1 to 7:3. Produced since 1954, ENDO was used worldwide at 11–13 kt year^−1^ between 1996 and 2004 (Weber et al. 2010). Endosulfan sulfate (ENSUL) is a persistent bioaccumulative metabolite. ENDO was added to the Stockholm Convention in 2011, with exemptions allowing some continued applications (UNEP 2014). Usage was limited in the HELCOM countries consisting of 110 kg in Finland in 2000 and minor amounts thereafter (HELCOM 2009). ENDO-I concentrations in arctic air were stable from 1993 to the mid-2000s, but a decrease was noted from 2006 to 2009 (Hung et al. 2013). Other currently used pesticides (CUPs) found in arctic regions include dacthal (DAC, chlorthal dimethyl), chlorpyrifos (CPF), chlorothalonil, dicofol, pentachloroanisole, pentachloronitrobenzene, and trifluralin (Hoferkamp et al. 2010; Zhong et al. 2012; Hung et al. 2013; Zhang et al. 2013), and even more CUPs were reported in air over the North Sea (Mai et al. 2013).

Atmospheric sources are estimated to dominate inputs to the Baltic for polychlorinated dibenzo-*p*-dioxins and dibenzofurans (PCDD/Fs) (Wiberg et al. 2009; Assefa et al. 2014), HCB, and polychlorinated biphenyls (PCBs) (Wiberg et al. 2009). Chlorinated pesticides in the Baltic are probably also derived largely from atmospheric deposition, but there are few data to corroborate this hypothesis, especially in northern Sweden. HCHs, DDTs, CHLs, and ENDOs in air and deposition are monitored at Environmental Monitoring and Evaluation Program (EMEP) stations in southern Sweden and arctic Finland (Hansson et al. 2006), and short-term campaigns have been conducted in northern Sweden (Newton et al. 2014).

### Natural brominated compounds

Many halogenated natural products (HNPs) are biosynthesized in the Baltic by macroalgae, phytoplankton, and sponges. These include halomethanes containing bromine, chlorine, and iodine (Orlikowska and Schulz-Bull 2009), bromophenols (BPs) and their transformation products BAs, polybrominated dibenzo-*p*-dioxins (PBDDs), and hydroxylated and methoxylated polybrominated diphenyl ethers (OH-BDEs, MeO-BDEs) (Unger et al. 2009; Löfstrand et al. 2010; Arnoldsson et al. 2012). Biosynthesis of MeO-BDEs and OH-BDEs may surpass formation through metabolism of anthropogenic PBDEs (Guitart et al. 2011). PBDDs contribute to toxic equivalents (TEQ) from PCDD/Fs and dioxin-like PCBs (van der Berg et al. 2013). The TEQ from PBDDs in seafood from the Swedish west coast and Baltic Proper are close to or exceed the European Union maximum residue limits in food for PCDD/Fs (Haglund et al. 2007). MeO-BDEs, OH-BDEs, and 2,4,6-tribromophenol (2,4,6-TBP) cause hormone-disrupting effects (Suzuki et al. 2008; Haldén et al. 2010; Hu et al. 2011; Wiseman et al. 2011). Methylation of BPs to BAs renders them non-ionic and easily volatilized. Higher levels of BAs in marine air occur near productive and upwelling regions of the North and South Atlantic (Führer and Ballschmiter 1998; Pfeifer and Ballschmiter 2002), and they have been measured in the Canadian Arctic (Wong et al. 2011) and coastal Norway (Melcher et al. 2008). Recently, we reported BAs in Baltic seawater and volatilization from Bothnian Bay (Bidleman et al. 2014).

## Experimental investigations

Surveys of chlorinated pesticides and BAs in surface water (0–5 m) were made in the Gulf of Bothnia (Fig. 1) during spring-summer of 2011–2012 and occasionally through ice in winter. One expedition to determine BAs from south to north in the Baltic was undertaken in September 2013 (Bidleman et al. 2014). Water was passed through glass fiber filters (GFFs), and the dissolved components were trapped on resin cartridges. “Passive” air samplers containing polyurethane foam (PUF) disks were deployed for 3–4 months at Holmön, an island in the “Quark” between Bothnian Bay and Bothnian Sea, from July 2011 to August 2012, and at Svartberget within Krycklan Catchment, 60 km from Bothnian Bay, from July 2011 to May 2012 (Fig. 1). “Pumped” air samples were collected at Holmön and on one expedition in the Gulf by drawing air through GFFs followed by PUF traps. Other pumped samples were taken at EMEP stations Råö, Sweden, Aspvreten, Sweden, and Pallas, Finland. “Bulk” (precipitation + dry particle) deposition samples were collected at Krycklan and Abisko, Sweden, from October 2009 to November 2010 (Newton et al. 2014) and at EMEP stations from January 2010 to December 2011. Sampling and analytical methods, quality control, and air–sea exchange calculations are given in Electronic Supplementary Material (S), Tables S1–S3.

## Results

Results presented here are new for pesticides in seawater, air at Holmön and Krycklan, and air and bulk deposition at EMEP stations. Previously reported bulk deposition measurements at Krycklan and Abisko (Newton et al. 2014) and air–water exchange of BAs in Bothnian Bay (Bidleman et al. 2014) are included.

### Pesticides and bromoanisoles in Gulf of Bothnia air and surface water

**Fig. 2:**
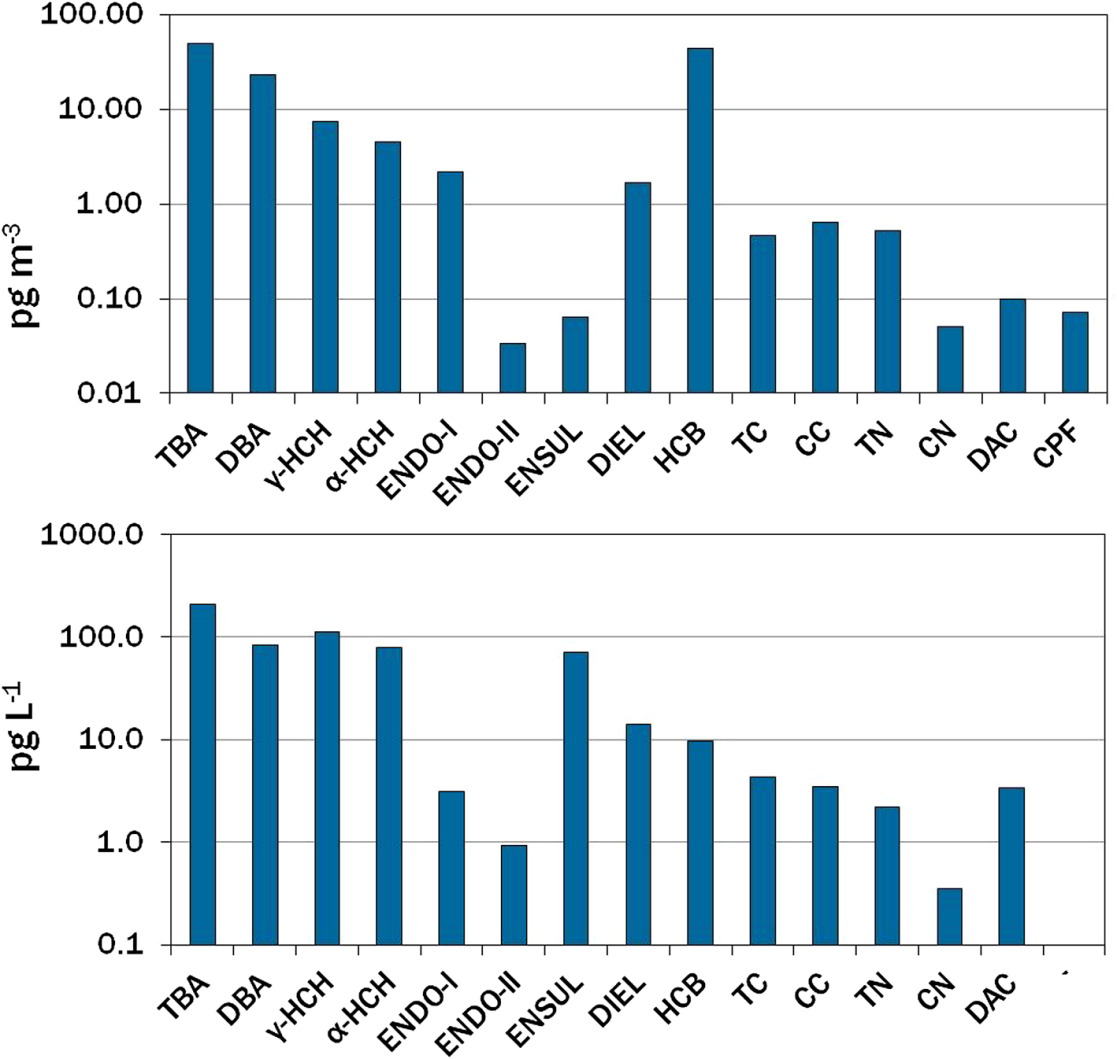
Concentrations of halogenated compounds in air (*top*) and Gulf of Bothnia surface water (*bottom*). CPF in water was <LOD. Numerical data are given in Electronic Supplementary Material, Tables S4 and S5. Compound abbreviations are in Table S1

Halogenated compounds in seawater and air are reported in Tables S4 and S5 and Fig. 2. Most abundant in water were 2,4,6-TBA, 2,4-DBA, HCHs, and the metabolite ENSUL, and at 1–2 orders of magnitude lower were 2,6-DBA, DIEL, HCB, CHL compounds (*trans*- and *cis*-chlordane, *trans*- and *cis*-nonachlor, TC, CC, TN, and CN), ENDO-I, ENDO-II, and DAC. CPF was below the limit of detection (LOD). Metabolites heptachlor *exo*-epoxide (HEPX) and *p*,*p*′-DDE (DDE) were quantified in air, but not in water due to chromatographic interferences.

Pesticide levels in air from passive sampling were higher in summer-fall (SF) than winter-spring (WS) for some compounds, while others did not differ seasonally or were lower in the warm period. The overall SF/WS ratio was 1.5 (Table S5). Concurrent passive air samples were obtained at Holmön and Krycklan during three periods (Table S5) for ENDO-I, HCB, TC, CC, TN, CN, and HEPX. A paired *t* test showed significant differences at *p* < 0.05 for HCB and CN and near-significant differences at *p* < 0.1 for TC and CC, but not for TN, HEPX nor ENDO-I. HCB and CHLs averaged, respectively, 55 and 73–88 % higher at Holmön, which suggests that proximity to water may have influenced air concentrations. Net volatilization was estimated for CHLs in summer and winter and for HCB in summer, whereas ENDO-I was depositing in all seasons (see below). BAs were identified in passive air samples from Holmön and Krycklan, but were too volatile to be trapped quantitatively, and results from pumped samples were used for exchange estimates (Bidleman et al. 2014).

Relative abundances in water and air were similar, though with some differences (Fig. 2). HCB and 2,4,6-TBA were most abundant in air, yet HCB was present at only moderate levels in water. ENDO-I dominated the group in air, reflecting the higher proportion of ENDO-I in the technical mixture, its greater volatility compared to ENDO-II, and possible isomerization of ENDO-II to ENDO-I (Weber et al. 2010). ENDO-II and ENSUL were usually below detection in passive air samples and found at only 1.5–3 % of ENDO-I levels in pumped air samples. In contrast, ENSUL in seawater exceeded ENDO-I and ENDO-II by 30–300 times.

The southern and northern Baltic are influenced by different air masses. During winter 2006–2007 sampling campaigns at Aspvreten and Pallas, 62 % of air transport to Aspvreten came from the SW-E and 37 % from the NW-NNE. PCDD/F levels (particulate + gaseous) at Aspvreten were 8 times higher than those at Pallas, where only 32 % of air transport came from the SW-ESE and 62 % from the W-NE (Sellström et al. 2009). Air pathways differ even within the Bothnian Bay catchment. Backward trajectories obtained during 2009–2010 deposition measurement campaigns showed that both Abisko and Krycklan were influenced mainly by W-NE transport, but Krycklan received greater contributions from southerly sectors (Newton et al. 2014). Air concentrations of pesticides at Holmön-Krycklan were similar to those reported at the three EMEP monitoring stations, Svalbard, and the Canadian Arctic (Table S6), which suggests no large gradients in background air concentrations.

### Air–sea gas exchange of pesticides in Bothnian Bay

**Fig. 3:**
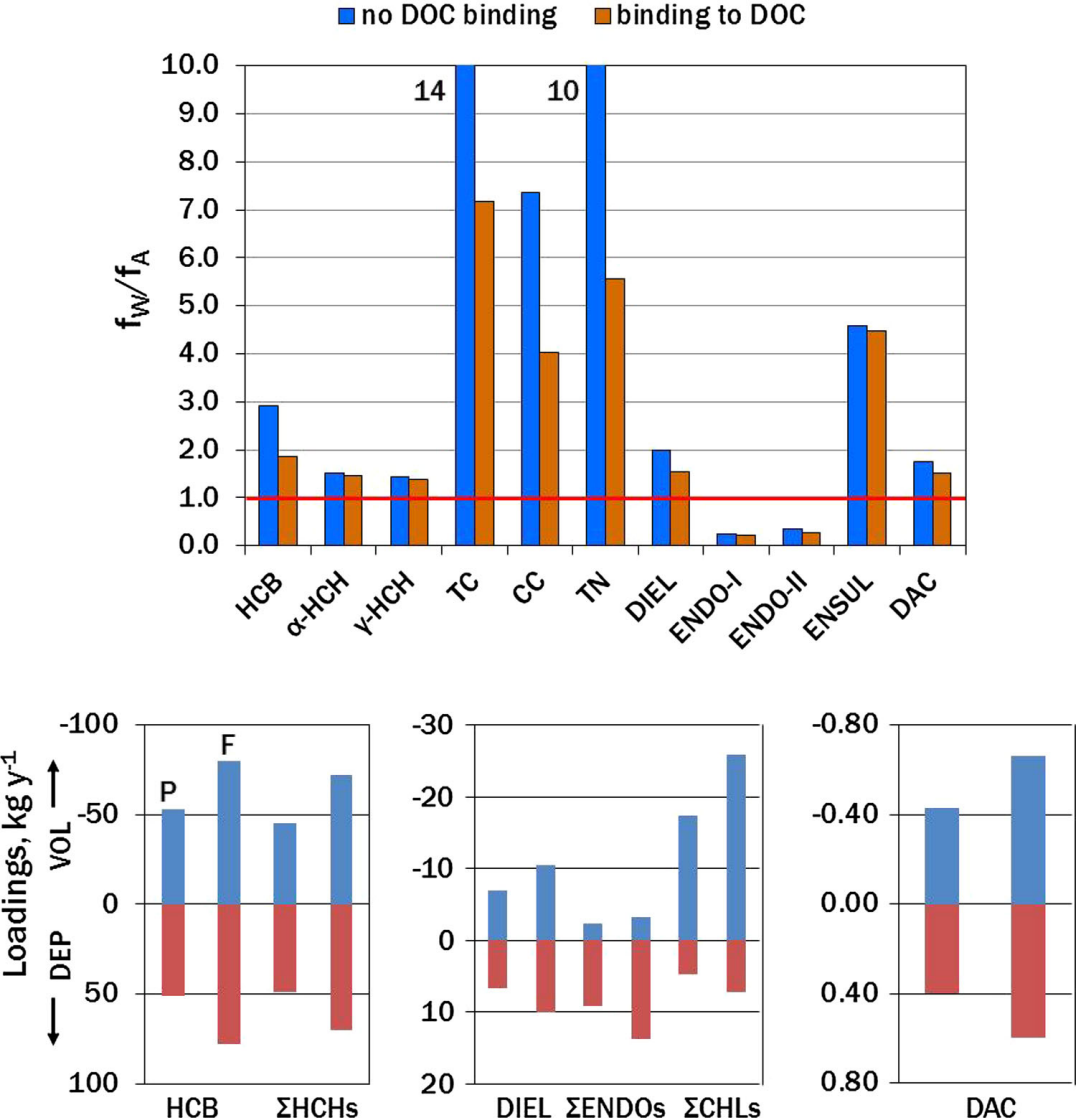
Gas exchange of pesticides in Bothnian Bay. *Top* Water/air fugacity ratios (*f*_W_ */f*_A_) at water and air temperatures of 15°C. Net deposition *f*_W_ */f*_A_ < 1, equilibrium *f*_W_ */f*_A_ = 1 (*red line*), and net volatilization *f*_W_ */f*_A_ > 1. Estimates are made assuming that the compounds are freely dissolved (*blue*) or partly sorbed to dissolved organic carbon (DOC) (*brown*). *Bottom* Gas deposition (*positive*) and volatilization (*negative*) loadings to Bothnian Bay (kg year^−1^) under the present situation (P) of water and air concentrations (Tables S4, S5), seasonal temperatures, and 4 months of ice cover, during which no exchange takes place; and a future mid- to late twenty-first century scenario (F) with increased water and air temperatures and no ice cover. Note that both deposition and volatilization loadings are increased in the future scenario, largely to the loss of ice. Temperatures and results of gas transfer calculations are given in Table S7

The saturation state of POPs in seawater with respect to air is described by the water/air fugacity ratio, *f*_W_*/f*_A_, where fugacity has units of Pascals. The *f*_W_*/f*_A_ = 1 at equilibrium. Oversaturation of water and net volatilization corresponds to *f*_W_*/f*_A_ > 1, while *f*_W_*/f*_A_ < 1 indicates undersaturation and net deposition. Fugacity ratios were calculated from annual average concentrations in water and air (Tables S4, S5) using Henry’s law constants at the monthly surface water temperature (Tables S3, S7). Results are displayed in Fig. 3 for situations in which the pesticides are freely dissolved in water or partly bound to dissolved organic carbon (DOC) (Ripszam and Haglund 2015). Percentages of freely dissolved compounds ranged from >90 % for HCHs and ENSUL, 75–85 % for DIEL, ENDOs, and DAC, and 50–65 % for HCB and CHLs. The reduction in *f*_W_*/f*_A_ caused by sorption to DOC was greater for the more hydrophobic compounds (Fig. 3).

The 95 % confidence intervals (95 % CI) were calculated for *f*_W_ and *f*_A_ (ESM). If these did not overlap, the exchange was judged as significant net volatilization or deposition, depending on which fugacity was greater. If the 95 % CIs overlapped, the exchange was not significantly different from equilibrium. Volatilization was estimated in July for HCB, α-HCH, TC, CC, TN, DIEL, DAC, and ENSUL. ENDO-I and ENDO-II were depositing, while γ-HCH was at equilibrium. In January, deposition was estimated for all compounds except TC, TN, and ENSUL (volatilization) and CC (equilibrium). CPF in water was <LOD, and HEPX and DDE were not determined in water (see above). Only gas-phase deposition was evaluated for these compounds.

Deposition and volatilization fluxes (*F*_DEP_, *F*_VOL_, mass area^−1^ time^−1^) and gas exchange loadings for Bothnian Bay (Table S7; Fig. 3) were estimated from the two-film model (ESM) assuming (1) the present situation of air and water temperatures (Table S7), average pesticide concentrations during 2011–2012 (Tables S4, S5), and 4 months of ice cover during which no exchange takes place; and (2) a future scenario in 2069–2099 in which temperatures increase by 3–6 °C in air and 2–4 °C in surface water (Table S7), suggested by mean predictions from the ENSEMBLE program (Meier et al. 2012a), and no ice cover. Concentrations in air and water were assumed constant. Propagation of uncertainties in flux estimates followed the procedure for fugacities (ESM). RSDs for *F*_VOL_ ranged from 30 to 50 % for 2,6-DBA, HCB, α-HCH, ENDO-I, ENSUL, DIEL, TN, and CN; and from 60 to 85 % for 2,4-DBA, 2,4,6-TBA, γ-HCH, ENDO-II, TC, CC, and DAC. RSDs for *F*_DEP_ were 30–50 % for 2,4-DBA, HCB, α-HCH, γ-HCH, ENDO-I, DIEL, CC, TN, CN, and DAC; and 60–95 % for 2,4,6-TBA, ENDO-II, ENSUL, DDE, TC, HEPX, and CPF. These apply only to *random* uncertainties; systematic error can also arise depending on the wind speed profile used in models to calculate mass transfer coefficients. Flux estimates using 3-h averaged wind speed were about 20–40 % higher than those using monthly averaged wind speed (Bidleman et al. 2014).

Results are shown in Fig. 3 and Table S7, where fluxes have been converted to annual deposition (positive) and volatilization (negative) loadings (kg) to Bothnian Bay (38 000 km^2^). HCB, ΣHCHs (α- + γ-HCHs), DIEL, and DAC were close to steady state, and net loss was estimated for ΣCHLs (TC + CC + TN + CN). The ΣENDOs group (ENDO-I + ENDO-II + ENSUL) was depositing, despite *f*_W_*/f*_A_ > 1 indicating net volatilization of ENSUL (Fig. 3), because the much more abundant ENDO-I dominated this group in air. Fluxes increased by about 50 % in the future scenario (Fig. 3), partly due to higher temperatures but mostly from loss of ice cover, which opens the bay to four additional months of gas exchange.

### Precipitation and dry particle deposition of pesticides

Deposition fluxes (ng m^−2^ month^−1^) from integrated monthly (Råö, Aspvreten, Pallas) or bimonthly (Abisko, Krycklan) bulk sampling are summarized in Table S8. HCHs and CHLs were reported at all stations. ENDOs were measured only at EMEP stations, and bulk deposition of DIEL and HCB was not determined. Higher HCH fluxes were found at Abisko, which in 2009–2010 received greater air transport from the Barents and Norwegian seas compared to Krycklan. Revolatilization from these seas may have augmented atmospheric levels (Newton et al. 2014). Arithmetic and geometric means (AM and GM) of the individual station results are given in Table S8, and GMs were used for loading estimates.

### Total atmospheric loadings of pesticides

**Fig. 4:**
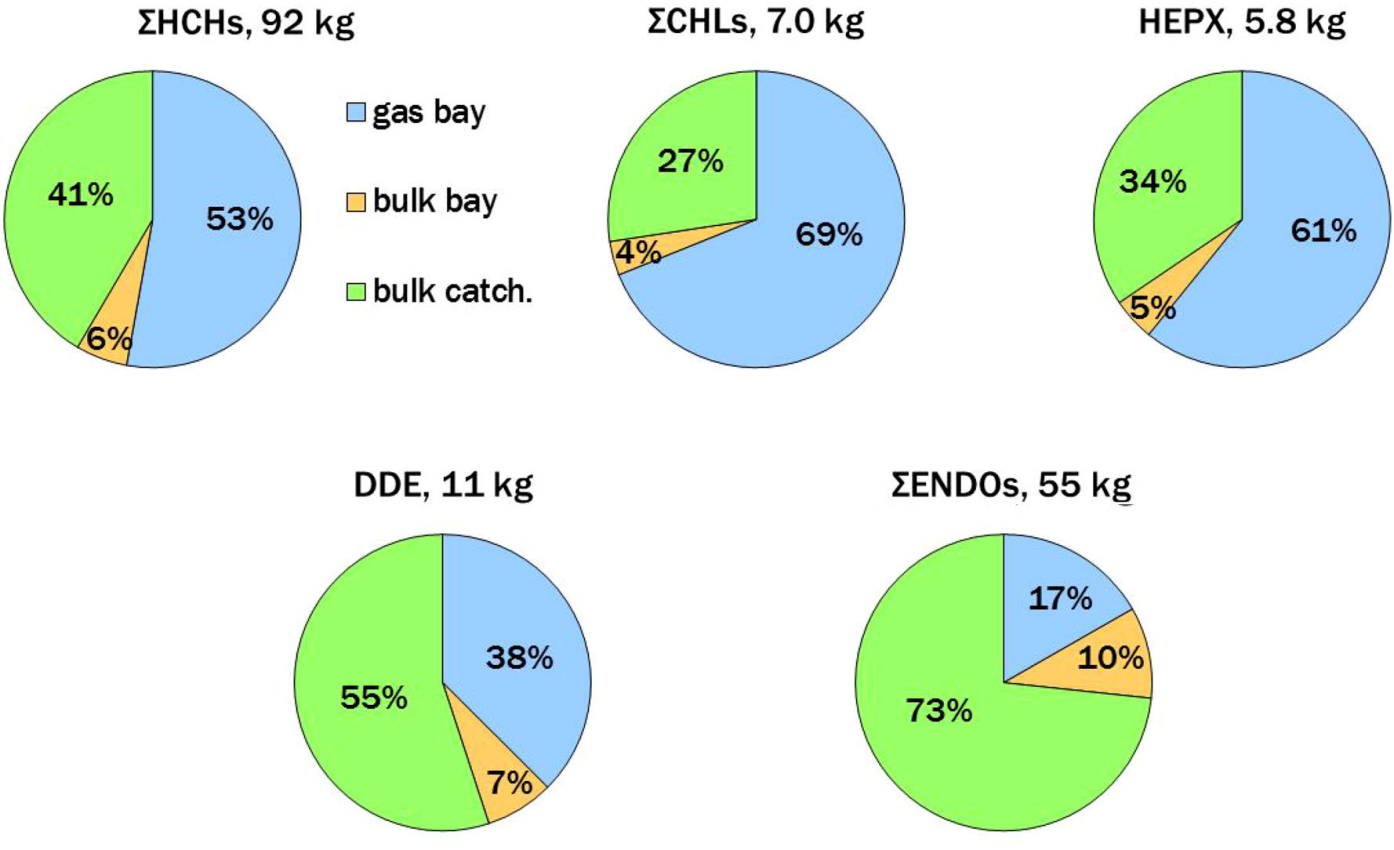
Total deposition loadings (kg year^−1^) of pesticides to the system of Bothnian Bay and its catchment. Loadings to the bay were estimated from the deposition component of gas exchange plus bulk (precipitation + dry particle) deposition (Tables S7, S8), whereas only bulk deposition was assessed for the catchment. The relative contributions of these processes are shown in the pie charts; e.g., of the 92 kg ΣHCHs atmospherically deposited, 59 % went into the bay (53 % by gas transfer and 6 % by bulk deposition) and 41 % went into the catchment by bulk deposition. These deposition loadings are offset by volatilization from the bay, resulting in net loadings (Table S9)

Atmospheric input to Bothnian Bay was assumed to occur by gas deposition (Table S7) plus bulk deposition (Table S8) to surface water, whereas bulk deposition alone delivered contaminants to the much larger bay catchment (280 000 km^2^). Deposition fluxes were multiplied by the relevant areas to estimate loadings as summarized in Table S9. Total annual deposition to Bothnian Bay + catchment was 92 kg ΣHCHs > 55 kg ΣENDOs > 11 kg DDE > 7.0 kg ΣCHLs ≈ 5.8 kg HEPX. The distribution between land and sea is shown in Fig. 4. For example, of the 92 kg ΣHCHs deposited to Bothnian Bay + catchment, the bay received 54 kg (48 kg by gas deposition and 5.5 kg by bulk deposition) and bulk deposition to the catchment was 38 kg. Atmospheric inputs of ΣHCHs and DDE were divided similarly between bay and catchment. The bay received the greater share of the total for ΣCHLs and HEPX, whereas the catchment received most of ΣENDOs. Volatilization removed −45, −17, and −2.4 kg year^−1^ of ΣHCHs, ΣCHLs, and ΣENDOs from Bothnian Bay, respectively (Tables S7, S9), such that the net loadings to the system were 46, −10, and 53 kg year^−1^ for these pesticide groups (Table S9). Net loadings could not be assessed for HEPX and DDE because water concentrations were not determined (see above).

### Air–sea exchange of bromoanisoles in Bothnian Bay

Exchange of BAs was estimated during the main algal production season from May to September (Bidleman et al. 2014). The BAs were oversaturated in surface water, with *f*_W_*/f*_A_ ranging from 3.4 to 7.6 for 2,4-DBA and 18 to 94 for 2,4,6-TBA, due to seasonally changing temperatures and concentrations. Volatilization removed −1360 kg of ΣBAs (2,4,6-TBA + 2,4-DBA + 2,6-DBA) in the 5-month period, an order of magnitude greater than volatilization of total pesticides (−125 kg year^−1^) (Tables S7, S9). The gas deposition pathway for BAs was minor, only 62 kg of 2,4,6-TBA + 2,4-DBA. Deposition of 2,6-DBA could not be assessed because its air concentrations were uncertain (Bidleman et al., 2014).

## Discussion

### Limits of the present study and historical perspective for HCHs

This study provides the first atmospheric loading estimates for CHLs and ENDOs to Bothnian Bay and its catchment, and updates the situation for HCHs. There are few complete mass budgets for pesticides, and a full accounting of inputs and losses was not made here. The relevant processes and compartments were identified in a POPCYCLING Baltic model of HCHs from 1970 to 2000, when HCHs were in common use and contamination was greater than today (Breivik and Wania 2002). HCHs dropped precipitously during the 1980s and early 1990s in response to declining emissions, and by 2000 accumulated amounts were an order of magnitude lower (Breivik and Wania 2002). Between 1990 and 2000, the Bothnian Bay catchment contained 65–70 % of total HCHs in the bay + catchment, resulting largely from uptake of atmospheric HCHs by forest soils, canopy, freshwater, and freshwater sediments (Breivik and Wania 2002). The forest canopy and deposited litter accumulate and release gaseous POPs (Nizzetto and Perlinger 2012; Nizzetto et al. 2014), whereas only precipitation + dry particle deposition to the catchment was estimated here (Table S9). Our study did not include advective flows to and from Bothnian Bay. In 1970–2000, the bay exported over twice the quantity of HCHs to the Bothnian Sea as received by the reverse flow (Breivik and Wania 2002).

Deposited chemicals enter streams and rivers, which flow into Bothnian Bay, but rates and efficiencies are poorly known. Association with DOC increases their mobility (Bergknut et al. 2011a), yet a study at Krycklan suggested that 96–99 % of atmospherically deposited PCBs and PCDD/Fs were retained within the landscape (Bergknut et al. 2011b). Whether the catchment is similarly retentive for less hydrophobic compounds such as HCHs, DAC, and ENDOs is an open question. In the period 1970–2000, rivers from the Bothnian Bay catchment delivered lower amounts of HCHs (10–20 %) to the sub-basin than by atmospheric deposition (Breivik and Wania 2002).

Little air and deposition monitoring of persistent chemicals has been conducted in northern Sweden. Air concentrations of chlorinated pesticides at Holmön–Krycklan were similar to those at EMEP and arctic monitoring stations (Table S6), but a greater range was found for bulk deposition (Table S8). Such disparity is expected from differences in precipitation type and amounts, temperatures, and air transport pathways. In addition to chlorinated pesticides and PCBs, the CUPs trifluralin and chlorothalonil, PBDEs, non-BDE flame retardants, and the flame retardant Dechlorane-Plus^®^ were identified in bulk deposition at Krycklan and Abisko (Newton et al. 2014). There is a need to monitor these and other chemicals of emerging concern at stations representative of the northern Baltic.

### Sources and pathways of bromoanisoles

Levels of BAs in Baltic air and water were similar to, or at the high end, of reports in other marine areas. This is surprising, because the Baltic has low salinity, especially in the north, and the availability of bromide is limited. Production of 2,4,6-TBP in the red alga *Ceramium tenuicorne* from the Baltic Proper increased nearly threefold between test salinities of 5–9‰ (Enhus et al. 2012). The ΣBAs (2,4,6-TBA + 2,4-DBA + 2,6-DBA) in surface water in September 2013 increased from 123 pg L^−1^ in the Gulf of Bothnia (2–5‰) to 200 pg L^−1^ in the Baltic Proper (6–7‰) to 460 pg L^−1^ in Kattegat (20‰) (Bidleman et al. 2014). Several factors may explain this gradient: insufficient bromide in the northern Baltic for optimum in situ formation, higher phytoplankton and BP production in the southern Baltic, and dilution with river water during advection northward.

Volatilization of BAs adds to the atmospheric bromine from emission of halomethanes, which are also produced by phytoplankton (Orlikowska and Schulz-Bull 2009). The flux of bromine from BA emissions in Bothnian Bay was 3 % of the bromine evasion from halomethanes in the southern Baltic. Higher BA concentrations in the southern Baltic (see above) could double this to 6 % (Bidleman et al. 2014). Other consequences of volatilization are removal from Bothnian Bay and long-range atmospheric transport. An estimate of BA residence time in the bay is about 50–70 days (Bidleman et al. 2014).

### Climatic factors relevant to Bothnian Bay

The potential influence of climate change on the cycling and fate of persistent chemicals has been reviewed by Kallenborn et al. (2012). Increased emissions from primary sources and secondary release from storage reservoirs can be expected. Processes likely to be affected are transport and distribution, persistence, bioavailability, and ecotoxicity. Here, climatic factors were investigated in a limited way by estimating the increased gas exchange of pesticides to Bothnian Bay due to loss of ice cover and rising temperature (Table S7; Fig. 3), but no account was made for changes in air and water concentrations. Levels of chlorinated pesticides in air at remote monitoring stations appear to be stabilizing or rising slightly in recent years (Ma et al. 2009; Becker et al., 2012; Hung et al. 2013). For pesticides that have been recently banned (e.g., ENDO), decreasing air concentrations and loadings can be expected.

A shift is anticipated from a phytoplankton- to microbial-based food web in the northern Baltic coupled to increasing terrestrial runoff, and phytoplankton production in the south may increase due to rising temperature and nutrient loading (Meier et al. 2012b; Andersson et al. 2013, 2015). The potential for volatilization is decreased by sorption to DOC (Fig. 2) and to particulate organic carbon (POC). Higher phytoplankton productivity increases POC, which by sinking acts as a “pump” to remove hydrophobic compounds from surface water (Josefsson et al. 2011; Nizzetto et al. 2012). Microbial biomass also provides surface for sorption of chemicals. These processes, plus higher degradation rates in warmer waters (“degradative pump”), could facilitate the air-to-sea pathway (Galbán-Malagón et al. 2013). Gas exchange rates increase with the square or cube of wind speed (ESM), and higher gust winds are predicted for the northern Baltic (Meier et al. 2012a).

Deposition to the terrestrial environment and subsequent runoff into the bay will be affected by changes in air concentrations, structure, and extent of the boreal forest and its soils, and precipitation amounts (Nizzetto and Perlinger 2012). A slight increase in HCB and PCB deposition to northern Swedish forests has been predicted from expected higher wintertime precipitation (Kallenborn et al. 2012).

The above comments also apply to BAs and other HNPs, with additional considerations. We have rudimentary knowledge of the variation in biosynthesis among algal species, seasonal and spatial distributions across the Baltic, and effects of salinity and DOC. Greater discharge of freshwater and DOC into the northern Baltic may reduce bromide availability and phytoplankton, thereby reducing HNPs. On the other hand, HNPs may increase due to reaction of hypobromite with DOC (Lin and Manley 2012) and by bacterial synthesis (Agarwal et al. 2014). Greater phytoplankton production in the southern Baltic may increase yields of HNPs, which to some degree would be advected to the northern basins.

## Conclusions

This initial study of chlorinated pesticides and BAs provides baseline information for future investigations of climate change on biogeochemical cycles in the northern Baltic Sea and its catchment. The catchment–sea link is a key to understand delivery of “indirect” atmospheric deposition via runoff, but remains not well understood. Loading estimates are also limited by lack of air and deposition monitoring in northern Sweden for legacy POPs as well as chemicals of emerging concern. Most air–sea exchange studies for POPs have been done in temperate and polar oceans where DOC concentrations are low. The Baltic is special with regard to its high level of DOC, which may increase further with changing climate. The role of DOC sources (e.g., autochthonous vs. allochthonous) and functionalities (e.g., more or less aromaticity) in sequestering chemicals of differing polarities merits further investigation, as does the role of other air–sea “pumping” mechanisms such as sinking POC and degradation of chemicals in the water column. Lessons learned from BAs are that their distribution is seasonally and spatially variable, and better understanding of BPC synthesis and biogeochemical pathways is needed. Despite limitations, a model of potential climate change impacts on HNP production and cycling and would sharpen the focus for future research.

## Electronic supplementary material

Supplementary material 1 (PDF 217 kb)
