# Supplementary material for: Atmospheric pathways of chlorinated pesticides and natural bromoanisoles in the northern Baltic Sea and its catchment
Source: Ambio. 2015 May 28;44(Suppl 3):472–83. doi: 10.1007/s13280-015-0666-4 (PMC4447703; doi:10.1007/s13280-015-0666-4)
Supplement: Supplementary file 1 — Supplementary material 1 (PDF 217 kb) [file 13280_2015_666_MOESM1_ESM.pdf]

**AMBIO**

Electronic Supplementary Material

This supplementary material has not been copy edited by the publisher or the editorial office.

Title: **Atmospheric pathways of chlorinated pesticides and natural bromoanisoles in the northern Baltic Sea and its catchment**

**Authors:** Terry Bidleman, Kathleen Agosta, Agneta Andersson, Eva Brorström-Lundén,
Peter Haglund, Katarina Hansson, Hjalmar Laudon, Seth Newton, Olle Nygren, Matyas Ripszam, Mats Tysklind, Karin Wiberg

**Table of contents**


| |
|---|
| Sampling locations and methods |
| Analytical methods |
| Quality control |
| Air-sea gas exchange |
| Uncertainty analysis and discussion |
| Table S1. Ions monitored for GC-ECNI-MS analysis |
| Table S2. Recoveries of labeled surrogate compounds (%) |
| Table S3. Henry's law constants at 25 $^{o}$C and parameters of log $H = m/T + b$ |
| Table S4. Organohalogen compounds in Gulf of Bothnia surface water, pg $L^{-1}$ |
| Table S5. Organohalogen compounds in passive and pumped air samples, pg $m^{-3}$ |
| Table S6. Comparison of mean pesticide concentrations in air at Holmön-Krycklan (H-K) and at air monitoring stations, pg $m^{-3}$. |
| Table S7. Temperatures for flux estimates and gas exchange loadings to Bothnian Bay |
| Table S8. Bulk deposition fluxes[a], ng $m^{-2}$ $month^{-1}$ |
| Table S9. Atmospheric loadings of halogenated compounds to Bothnian Bay and catchment, kg. |
| References |


**Sampling locations and methods**

Surveys of chlorinated pesticides and bromoanisoles (BAs) in surface seawater (0-5 m) were made in the Gulf of Bothnia (Figure 1, main paper) during spring-summer of 2011-2012 and occasionally through ice in winter. One expedition to determine BA concentrations from south to north in the Baltic was undertaken in September, 2013. Sampling dates and locations are reported in Bidleman et al. (2014). Water was passed through glass fiber filters (GFFs) and the dissolved components from 40 L were sorbed onto XAD-2 resin for

chlorinated pesticides (Jantunen and Bidleman, 1998; Jantunen et al., 2004) and BAs, or from 5 L onto ENV+ resin cartridges for BAs only (Bidleman et al., 2014).

Two types of air samples were collected. "Passive" air samplers contained polyurethane foam (PUF) disks. These were sheltered within stainless steel enclosures to protect them from high wind and precipitation (Shoeib and Harner, 2002; Bohlin et al., 2014). Duplicate samplers were deployed for 3-4 months to collect chlorinated pesticides at Holmön (63.792N, 20.839E), an island in the "Quark" or nexus between Bothnian Bay and Bothnian Sea from July 2011 to August 2012, and at Svartberget within Krycklan Catchment (64.233N, 19.767E), 60 km from Bothnian Bay, from July 2011 to May 2012 (Figure 1). BAs were too volatile to be efficiently trapped by the PUF disks over this time. We estimated sampling rates for the pesticides from the loss rates of spiked depuration compounds PCBs 19 and 54 and the PCB- or pesticide-specific octanol-air partition coefficients (Gouin et al., 2005; Bohlin et al., 2014). Sampling rates averaged 2.2-3.1 $m^3 d^{-1}$ for the more volatile HCB and HCHs, and 3.7-4.1 $m^3 d^{-1}$ for chlordane compounds (TC, CC, TN and CN), DIEL, ENDO-I, ENDO-II, DAC and CPF (abbreviations in Table S1). A critical evaluation of PUF-based passive collectors found a mean sampling rate of 3.5 ± 1.9 $m^3 d^{-1}$ for all classes of semivolatile compounds (Bohlin et al., 2014).

"Pumped" air samples for pesticides were collected at Holmön and Environmental Monitoring and Evaluation Program (EMEP) stations Råö (Sweden, 57.383N, 11.917E), Aspvreten (Sweden, 58.806N, 17.388E), and Pallas (Finland, 68.159N, 24.040E) (Hansson et al., 2006). These were taken by drawing air through a GFF followed by a PUF trap at approximately 0.5 $m^3$ per min over 24-48 h (Jantunen et al., 2004; Hansson et al., 2006). Pumped air samples were also taken from shipboard in the Gulf of Bothnia during one week of May 2012. Lower air volumes were sampled over 2-4 h to avoid breakthrough losses from the PUF traps for 2,4-dibromoanisole (2,4-DBA) and 2,4,6-tribromoanisole (2,4,6-TBA), but breakthrough was found for the more volatile 2,6-DBA and its air concentration was estimated as a lower limit (Bidleman et al., 2014).

"Bulk" atmospheric deposition samples (precipitation + dry particle deposition) were collected at Abisko, Sweden (68.333N, 19.050E) and Krycklan from October 2009 to November 2010 using a glass funnel and Amberlite IRA 743 resin cartridge (Newton et al.,

2014) and at the EMEP stations from January 2010 to December 2011 using a Teflon®-coated funnel and PUF trap (Hansson et al., 2006).

**Analytical methods**

Since the main purpose of the study was to investigate air-sea gas exchange, only air and seawater sample components representing the "gas phase" (PUF traps) and the "dissolved phase" (XAD-2 or ENV+ cartridges) were analyzed. After extraction with organic solvents and cleanup, analysis was conducted by capillary gas chromatography – low resolution mass spectrometry (GC-LRMS) with electron impact (EI) ionization for BAs (Bidleman et al., 2014) and electron capture negative ionization (ECNI) for chlorinated pesticides (Table S1). Chlorinated pesticides were determined by GC-high resolution mass spectrometry (GC-HRMS) in bulk deposition samples from Abisko and Krycklan (Newton et al., 2014), and by GC-electron capture detection (GC-ECD) for most pesticides (GC-LRMS for ENDOs) in air and bulk deposition samples from EMEP (Hansson et al., 2006).

**Quality control**

Recoveries of chlorinated pesticides were monitored by adding $^{13}$C- or $^{2}$H-labeled compounds. The spikes were added to water samples before sorption onto XAD-2 or ENV+, and to the PUFs of passive or pumped air samples before extraction in the laboratory. Recoveries of all surrogates from XAD-2 averaged 61 ± 27% (n=112) and from PUFs 101 ± 27% (n=263) (Table S2). Recoveries of BAs were monitored with the surrogates $^{2}H_5$-2,4,6-TBA or $^{13}C_6$-γ-HCH, and results are reported in Bidleman et al. (2014). Adjustments for recoveries were made on an individual sample basis.

Limits of detection (LODs) were determined by analyzing blank sampling media (PUF, XAD-2, ENV+); the LOD was calculated as the mean blank + 3*s.d. In many cases no chromatographic peak was found at the retention time of the analyte, and the LOD was estimated as the instrumental detection limit (IDL), determined by integrating baseline "noise". LODs are listed in Table S4 for water and Table S5 for air.

Duplicate passive samplers were deployed in seven periods, with a mean difference of 12% in collected quantities across all compounds. A mean difference of 18% was found for duplicate determinations of BAs in water (n=6) (Bidleman et al., 2014).

Quality control procedures for atmospheric deposition at Abisko and Krycklan (Newton et al., 2014) and at EMEP stations (Hansson et al., 2006) is given in these respective publications.

**Air-sea gas exchange**

The potential for exchange between gaseous and dissolved species was expressed by the water/air fugacity ratio, where $f$ has units of Pascals (Pa):

$$f_W = C_W H \qquad (1)$$

$$f_A = C_A R T_A \qquad (2)$$

$$f_W/f_A = C_W H / C_A R T_A \qquad (3)$$

$C_W$ and $C_A$ are concentrations in water and air (mol $m^{-3}$), $H$ is the Henry's law constant at the water temperature (Pa $m^3$ $mol^{-1}$), $T_A$ is air temperature (K), and $R$ = 8.31 Pa $m^3$ $mol^{-1}$ $K^{-1}$. Air-water equilibrium is indicated by $f_W/f_A = 1$, whereas $f_W/f_A < 1$ or $f_W/f_A > 1$ indicate net deposition or net volatilization (Jantunen et al., 2004; Bidleman et al., 2014).

Henry's law constants at 25 $^{o}$C were selected from literature reports (Table S3). Thermodynamically consistent "final adjusted values" (Muir et al., 2004; Xiao et al., 2004; Shen and Wania, 2005) were used wherever possible. $H$ at 25 $^{o}$C were adjusted to the temperature of Baltic seawater using the relationship:

$$\text{Log } H = m/T + b \qquad (4)$$

Slopes ($m$) of eq 4 were obtained from references in Table S3, and $b$ were calculated using these slopes and $H$ at 25 $^{o}$C. Exchange estimates for ENSUL are speculative because its Henry's law constant at 25 $^{o}$C is known only approximately (see below), and the slope for ENDO-II was assumed.

Association of pesticides and BAs with dissolved organic carbon (DOC) renders them less available for air-water exchange. This factor was taken into account using the $K_{DOC} = C_{X,DOC}/C_{X,FREE}$ values determined by Ripszam et al. (2015), where $C_{X,DOC}$ and $C_{X,FREE}$ are concentrations of the substances bound to DOC and in freely dissolved form. A Baltic DOC concentration of 4.8 mg $L^{-1}$ was assumed (Bidleman et al., 2014).

Deposition, volatilization and net fluxes ($F_{DEP}$, $F_{VOL}$, $F_{NET}$, ng $m^{-2}$ $d^{-1}$) were estimated using the two-film gas exchange model, as described in the Supporting Information of Bidleman et al. (2014) for BAs, and extended here to chlorinated pesticides. We used equations from Wanninkhof and McGillis (1999) and Mackay and Yeun (1983) to estimate mass transfer coefficients (MTCs, m $s^{-1}$) for pesticides in the individual liquid (water, $k_L$) and gas (air, $k_G$) films as functions of wind speed at 10 m height ($U_{10}$, m $s^{-1}$), and dimensionless Schmidt numbers for $CO_2$ and the pesticides in water ($Sc_L/Sc_{L,CO2}$) and air ($Sc_G$):

$$k_L = 7.87x10^{-8} U_{10}^3 (Sc_L / Sc_{L,CO2})^{-0.5} \qquad (5)$$

$$k_G = 1x10^{-3} + 4.62x10^{-4} (6.1 + 0.63 U_{10})^{0.5} U_{10} Sc_G^{-0.67} \qquad (6)$$

Wind speeds at Holmön were obtained every three hours from the Swedish Meteorological and Hydrological Institute (SMHI). Three-hour mean $U_{10}$ were sorted into bins of 1 m $s^{-1}$ and plotted as frequency distributions, shown for May – September in Supporting Information of Bidleman et al. (2014) and extended to other months here. Wind speed weighted MTCs were calculated by multiplying the $k_L$ and $k_G$, calculated from $U_{10}$ at each bin's midpoint by the fractional bin frequencies and summed (Bidleman et al., 2014).

Details for calculating Schmidt number (kinematic viscosity of water or air divided by the molecular diffusivity of the compound in these media) are given for BAs in Bidleman et al. (2014) and extended here for chlorinated pesticides. Whereas diffusivities and kinematic viscosities are temperature dependent, $Sc_L/Sc_{L,CO2}$ and $Sc_G$ vary only slightly between 0 – 25 $^oC$ and among the different pesticides. Average values at 15 $^oC$ were used in flux calculations, $Sc_G = 3.13$, and $Sc_L/Sc_{L,CO2} = 3.40$.

Volatilization, deposition and net fluxes ($F$, mol $m^{-2}$ $d^{-1}$) were calculated with the series of equations below:

$$F_{VOL} = -D_{OL} f_W \qquad (7)$$

$$F_{DEP} = D_{OL} f_A \qquad (8)$$

$$F_{NET} = D_{OL}(f_A - f_W) \qquad (9)$$

$$D_{OL} = 86400 K_{OL} / H \qquad (10)$$

$$1/K_{OL} = 1/k_L + RT/Hk_G \qquad (11)$$

In these equations, $D_{OL}$ (mol m$^{-2}$ d$^{-1}$ Pa$^{-1}$) and $K_{OL}$ (m s$^{-1}$) are "overall" MTCs which account for resistance by both the water and air films, and 86400 = s d$^{-1}$. Deposition flux is positive and volatilization is negative.

**Uncertainty analysis and discussion**

Uncertainties (standard deviations, SD; relative standard deviations, RSD) in water and air fugacities were derived from the definitions in eq. 1 and 2 by:

$$(RSD\ f_W)^2 = (RSD\ C_W)^2 + (RSD\ H)^2 \qquad (12)$$

$$SD\ f_W = f_W \times (RSD f_W) \qquad (13)$$

$$SD\ f_A = SD\ C_A \qquad (14)$$

RSDs in $C_W$ and $C_A$ were obtained from the means and SDs in Tables S4 and S5. The RSD in $H$ was assumed to be 0.2, on the basis of experimental measurements (Sahsuvar et al., 2003; Cetin et al., 2006; Jantunen and Bidleman, 2006), although systematic non-random errors in reported values by different research groups may be considerably larger. We have tried to minimize these by selecting "final adjusted values" (FAVs) wherever possible (Muir et al., 2004; Xiao et al., 2004; Shen and Wania, 2005;). The Henry's law constant for ENSUL at 25$^{o}$C is only an approximate value obtained from vapor pressure/water solubility (Weber et al., 2010) and its uncertainty is likely to be greater than 20%.

The 95% confidence intervals (95% CI) for $f_W$ and $f_A$ were calculated from:

$$f_W \pm 95\%\ CI = f_W \pm t_{95} \times (SD\ f_W) \times N_W^{-0.5} \qquad (15)$$

$$f_A \pm 95\%\ CI = f_A \pm t_{95} \times (SD\ f_A) \times N_A^{-0.5} \qquad (16)$$

where $N_W$ and $N_A$ are the number of water and air samples. If these 95% confidence intervals did not overlap, the compound was judged to undergo significant net volatilization or deposition, depending on whether $f_W > f_A$ or $f_W < f_A$. If the 95% confidence intervals overlapped, the exchange was judged to be not significantly different from air-water equilibrium.

Based on this assessment, net volatilization of the following compounds was estimated in July: HCB, α-HCH, TC, CC, TN, DIEL, DAC and ENSUL. ENDO-I and ENDO-II were undergoing net deposition, while γ-HCH was at equilibrium. In January, net deposition was estimated for all compounds except TC, TN and ENSUL (net volatilization) and CC (equilibrium).

Since volatilization and deposition fluxes ($F_{VOL}$ and $F_{DEP}$, eq. 7 and 8) are functions of $f_W$ and $f_A$, propagation of errors follows eq. 12-14 if the MTC ($D_{OL}$) is considered invariant. Resulting RSDs for $F_{VOL}$ ranged from 30-50% for 2,6-DiBA, HCB, α-HCH, ENDO-I, ENSUL, DIEL, TN and CN; and 60-85% for 2,4-DBA, 2,4,6-TBA, γ-HCH, ENDO-II, TC, CC, and DAC. RSDs for $F_{DEP}$ were 30-50% for 2,4-DBA, HCB, α-HCH, γ-HCH, ENDO-I, DIEL, CC, TN, CN and DAC; and 60-95% for 2,4,6-TBA, ENDO-II, ENSUL, *p,p'*-DDE, TC, HEPX and CPF.

Variation in $D_{OL}$ (eq. 10) is related to uncertainties in $H$ and $K_{OL}$ (eq. 11). The 20% random uncertainty due to $H$ (see above) is likely minor compared to error in $K_{OL}$, which is calculated from the individual MTCs $k_L$ and $k_G$ (eq. 5 and 6). These MTCs are nonlinear functions of wind speed, $U_{10}$. In our previous study of BA air-sea exchange (Bidleman et al., 2014), $F_{VOL}$ was calculated using $K_{OL}$ derived from $k_G$ (eq. 6) and two relationships for $k_L$, eq. 5 for 3-hour averaged $U_{10}$ and another relationship for long-term (monthly) average winds:

$$k_L = 2.78x10^{-6}\left(1.09U_{10} - 0.333U_{10}^2 + 0.078U_{10}^3\right)\left(Sc_L / Sc_{L,CO2}\right)^{-0.5} \qquad (17)$$

$F_{VOL}$ of the BAs using the MTCs derived from monthly average $U_{10}$ were about 70-75% of those estimated from MTCs weighted for 3-hour mean $U_{10}$ (the approach taken in this paper, see above). This comparison was also made for the OCPs, with the result that the gas

exchange volatilization and deposition loadings presented in Table S7 (based on 3-hour wind speed-weighted MTCs) are about 20-40% higher than those calculated using monthly average $U_{10}$..

Uncertainties in the bulk deposition estimates were assessed by comparing the geometric mean (GM) and arithmetic mean (AM) of the five AMs from the deposition stations (Table S8 ). AMs were 2-37% larger than GMs for most compounds, but factors of 2-3 larger for the HCHs, due to the much higher deposition values found at Abisko.  The GM deposition estimates were used in this paper.

**Table S1. Ions monitored for GC-MS analysis[a].**

| | Quant. | Qual. | | Quant. |
|---|---|---|---|---|
| γ-hexachlorocyclohexane (γ-HCH) | 255 | 257 | $^{13}C_6$-HCB | 292 |
| α-hexachlorocyclohexane (α-HCH) | 255 | 257 | $^{13}C_6$-γ-HCH | 261 |
| endosulfan-I (ENDO-I) | 404 | 406 | $^{13}C_{10}$-HEPX | 398 |
| endosulfan-II (ENDO-II) | 404 | 406 | $^{13}C_{10}$-TN | 454 |
| endosulfan sulfate (ENSUL) | 386 | 388 | $^{13}C_{12}$-DIEL | 392 |
| dieldrin (DIEL) | 380 | 382 | $^{13}C_9$-ENDO-I | 413 |
| *p,p*'-DDE (DDE) | 318 | 316 | $^{13}C_9$-ENDO-II | 413 |
| hexachlorobenzene (HCB) | 284 | 286 | $^{13}C_{10}$-CPF | 322 |
| *trans* -chlordane (TC) | 410 | 412 | $^{2}H_{14}$-trifluralin (TFN) | 349 |
| *cis* -chlordane (CC) | 410 | 412 | $^{2}H_5$-2,4,6-TBA | 351 |
| *trans* -nonachlor (TN) | 444 | 446 | $^{13}C_{12}$-PCB105 (int. std.) | 338 |
| *cis* -nonachlor (CN) | 444 | 446 | | |
| heptachlor *exo* -epoxide (HEPX) | 388 | 386 | | |
| dacthal (chlorthal dimethyl) (DAC) | 332 | 330 | | |
| chlorpyrifos (CPF) | 313 | 315 | | |
| 2,4-dibromoanisole (2,4-DBA) | 266 | 264 | | |
| 2,6-dibromoanisole (2,6-DBA) | 266 | 264 | | |
| 2,4,6-tribromoanisole (2,4,6-TBA) | 344 | 346 | | |

a) Electron capture negative ion (ECNI) for pesticides and labeled PCB-105, electron impact (EI) for bromoanisoles.

**Table S2. Recoveries of labeled surrogate compounds (%)**

| | air (passive and active) | | | water | | |
|---|---|---|---|---|---|---|
| | **mean** | **SD** | **N** | **mean** | **SD** | **N** |
| $^{13}C_6$-HCB | 68 | 15 | 19 | | | |
| $^{13}C_6$-γ-HCH | 100 | 18 | 40 | 60 | 14 | 14 |
| $^{13}C_{10}$-HEPX | 97 | 24 | 39 | 49 | 14 | 14 |
| $^{13}C_{10}$-TN | 91 | 21 | 40 | 45 | 15 | 14 |
| $^{13}C_{12}$-DIEL | 94 | 23 | 39 | 69 | 30 | 14 |
| $^{13}C_9$-ENDO-I | 99 | 20 | 37 | 42 | 17 | 14 |
| $^{13}C_9$-ENDO-II | 107 | 39 | 40 | 51 | 33 | 14 |
| $^{13}C_{10}$-CPF | 116 | 37 | 28 | 97 | 41 | 14 |
| $^{2}H_{14}$-TFN | | | | 77 | 35 | 14 |
| All surrogates | 101 | 27 | 263 | 61 | 27 | 112 |

**Table S3. Henry's law constants at 25°C and parameters of log *H* = *m/T* + *b***

| | *H* | log *H* | | | Reference[a] | | |
|---|---|---|---|---|---|---|---|
| | 25 °C | 25 °C | *m* | *b* | *H* , 25 °C | *m* | Comments |
| γ-HCH | 0.31 | -0.509 | -3117 | 9.94 | 1 | 1 | FAV[b] |
| α-HCH | 0.74 | -0.131 | -3099 | 10.26 | 1 | 1 | FAV |
| ENDO-I | 0.71 | -0.152 | -1784 | 5.83 | 3 | 2 | FAV |
| ENDO-II | 0.045 | -1.347 | -1647 | 4.28 | 3 | 2 | FAV |
| ENSUL | 0.015 | -1.824 | -1647 | 3.70 | 5 | ENDO-II[c] | |
| DIEL | 1.1 | 0.046 | -2462 | 8.30 | 3 | 2 | FAV |
| HCB | 65.0 | 1.813 | -2637 | 10.66 | 3 | 2 | FAV |
| TC | 6.9 | 0.837 | -2302 | 8.56 | 3 | 2 and 4 | FAV |
| CC | 5.8 | 0.760 | -2190 | 8.10 | 3 | 2 and 4 | FAV |
| TN | 12.1 | 1.084 | -2748 | 10.30 | 2 | 2 and 4 | |
| DAC | 0.23 | -0.638 | -2363 | 7.29 | 6 | average[d] | FAV |

a) 1. Xiao et al., 2004; 2. Cetin et al., 2006; 3. Shen and Wania, 2005;
4. Jantunen and Bidleman, 2006; 5. Weber et al., 2010; 6. Muir et al., 2004.

b) FAV: "Final adjusted value" at 25 °C were derived from combining physicochemical properties and partitioning relationships, and adjusting these to minimize errors and achieve thermodynamic consistency.

c) The slope for ENDO-II was assumed.

d) The average of other slopes in the table was assumed.

**Table S4. Organohalogen compounds in Gulf of Bothnia surface water, pg L$^{-1}$.**

| Compound[a] | Mean | SD | N | LOD[b] |
|---|---|---|---|---|
| 2,4,6-TBA[c] | 209 | 164 | 17 | 2.6-10 |
| 2,4-DBA[c] | 83 | 47 | 17 | 0.8-4.0 |
| 2,6-DBA[c] | 14 | 7.0 | 12 | 0.8-4.0 |
| γ-HCH | 112 | 67 | 15 | 3.0 |
| α-HCH | 80 | 21 | 15 | 3.7 |
| ENDO-I | 3.1 | 0.87 | 9 | 0.32 |
| ENDO-II | 0.93 | 0.77 | 8 | 0.44 |
| ENSUL | 72 | 35 | 14 | 2.3 |
| DIEL | 14 | 4.2 | 14 | 2.0 |
| HCB | 9.7 | 4.6 | 14 | 1.0 |
| TC | 4.3 | 3.5 | 14 | 0.83 |
| CC | 3.5 | 1.9 | 13 | 0.52 |
| TN | 2.2 | 1.1 | 13 | 0.20 |
| CN | 0.35 | 0.13 | 9 | 0.14 |
| DAC | 3.4 | 1.9 | 14 | 0.58 |
| CPF | ND[d] | | 11 | 1.8 |

a) Abbreviations in Table S1.

b) LOD, pg L$^{-1}$. Limit of detection. Assumes 40 L sample volume for pesticides, 5-40 L for DBA and TBA.

c) Data from Bidleman et al., 2014.

d) Not detected, <LOD.

**Table S5. Organohalogen compounds in passive and pumped air samples, pg $m^{-3}$.**

| | Passive sampling | | | | | | | | | | |
|---|---|---|---|---|---|---|---|---|---|---|---|
| | Holmön, period | | | | Krycklan, period | | | | | | |
| | **1** | **2** | **3** | **4** | **1** | **2** | **3** | **mean** | **SD** | **N** | **LOD**[b] |
| Start | 27/07/2011 | 26/10/2011 | 22/01/2012 | 17/05/2012 | 08/07/2011 | 20/10/2011 | 22/01/2012 | | | | |
| Stop | 26/10/2011 | 22/01/2012 | 17/05/2012 | 11/08/2012 | 20/10/2011 | 22/01/2012 | 16/05/2012 | | | | |
| Days | 86 | 93 | 116 | 86 | 104 | 94 | 116 | | | | |
| **Compound**[a] | | | | | | | | | | | |
| γ-HCH | 3.9 | 3.9 | I[c] | 9.7 | 3.9 | 2.7 | 5.0 | 4.9 | 2.5 | 9 | 0.43 |
| α-HCH | 15 | 9.5 | I | 14 | 13 | 5.8 | 3.6 | 10 | 4.7 | 11 | 0.61 |
| ENDO-I | 3.4 | 2.7 | 3.3 | 2.4 | 4.2 | 1.2 | 2.5 | 2.8 | 0.95 | 13 | 0.090 |
| ENDO-II | *0.03*[d] | *0.03* | *0.03* | *0.03* | 0.066 | *0.03* | *0.03* | *0.03* | *0.03* | 7 | 0.059 |
| ENSUL | *0.04* | 0.27 | *0.04* | *0.04* | *0.04* | *0.04* | I | *0.04* | *0.04* | 6 | 0.080 |
| DIEL | 2.5 | 2.8 | I | I | 1.6 | 1.0 | 1.6 | 1.9 | 0.7 | 10 | 0.29 |
| DDE | 1.3 | 3.3 | I | I | 1.4 | 1.3 | 1.0 | 1.7 | 0.93 | 7 | 0.47 |
| HCB | 34 | 60 | 43 | 21 | 23 | 40 | 26 | 35 | 14 | 13 | 1.5 |
| TC | 0.22 | 0.42 | 0.35 | 0.23 | 0.15 | 0.19 | 0.23 | 0.26 | 0.095 | 13 | 0.040 |
| CC | 0.82 | 0.74 | 0.62 | 0.69 | 0.62 | 0.32 | 0.42 | 0.60 | 0.18 | 13 | 0.049 |
| TN | 0.69 | 0.72 | 0.63 | 0.65 | 0.62 | 0.26 | 0.44 | 0.57 | 0.16 | 13 | 0.027 |
| CN | 0.11 | 0.050 | 0.050 | 0.10 | 0.089 | 0.018 | 0.031 | 0.064 | 0.036 | 13 | 0.014 |
| HEPX | 1.8 | 1.1 | *0.2* | 2.5 | 1.5 | 0.46 | 0.56 | 1.2 | 0.83 | 11 | 0.43 |
| DAC | 0.12 | 0.055 | NA[e] | NA | 0.11 | 0.022 | NA | 0.077 | 0.046 | 8 | 0.007 |
| CPF | 0.030 | 0.12 | NA | NA | *0.014* | 0.030 | NA | 0.049 | 0.048 | 8 | 0.028 |

**Table S5. Organohalogen compounds in passive and pumped air samples, pg m$^{-3}$ (continued).**

| | Pumped sampling, Holmön & ship, May-Sept., 2011-2012 | | | | Passive + pumped air samples | | |
|---|---|---|---|---|---|---|---|
| | **mean** | **SD** | **N** | **LOD[b]** | **mean** | **SD** | **N** |
| **Compound[a]** | | | | | | | |
| 2,4,6-TBA[f] | 50 | 31 | 10 | 0.34 | 50 | 31 | 10 |
| 2,4-DBA[f] | 23 | 10 | 10 | 0.41 | 23 | 10 | 10 |
| γ-HCH | 4.2 | 1.1 | 12 | 0.43 | 4.5 | 1.8 | 21 |
| α-HCH | 5.4 | 1.9 | 16 | 0.61 | 7.3 | 3.3 | 27 |
| ENDO-I | 1.7 | 0.9 | 17 | 0.036 | 2.2 | 0.92 | 30 |
| ENDO-II | 0.036 | 0.024 | 9 | 0.024 | 0.033 | 0.027 | 16 |
| ENSUL | 0.079 | 0.071 | 9 | 0.032 | 0.063 | 0.061 | 15 |
| DIEL | 1.5 | 0.62 | 15 | 0.12 | 1.7 | 0.67 | 25 |
| DDE | 0.84 | 0.38 | 12 | 0.19 | 1.1 | 0.64 | 19 |
| HCB | 58 | 25 | 8 | 1.5 | 43.9 | 18.9 | 21 |
| TC | 0.63 | 0.45 | 17 | 0.016 | 0.47 | 0.34 | 30 |
| CC | 0.65 | 0.29 | 17 | 0.020 | 0.63 | 0.25 | 30 |
| TN | 0.47 | 0.19 | 17 | 0.011 | 0.51 | 0.18 | 30 |
| CN | 0.036 | 0.011 | 13 | 0.005 | 0.050 | 0.026 | 26 |
| HEPX | 0.68 | 0.27 | 13 | 0.17 | 0.90 | 0.59 | 24 |
| DAC | 0.11 | 0.057 | 14 | 0.003 | 0.098 | 0.053 | 22 |
| CPF | 0.088 | 0.051 | 12 | 0.011 | 0.072 | 0.050 | 20 |

a) Abbreviations in Table S1.

b) LOD for passive sampling, pg m$^{-3}$: Limit of detection = mean blank or IDL + 3*SD. Assumes volumes of 250 m$^3$ for HCB and HCHs, and 400 m$^3$ for others. LOD for active sampling, pg m$^{-3}$: Limit of detection = mean blank or IDL + 3*SD. Assumes volumes of 100 m$^3$ for 2,4-DBA and 2,4,6-TBA, 250 m$^3$ for HCB and HCHs, and 400 m$^3$ for others.

c) I = chromatographic interference, not quantified.

d) Numbers in italics: Estimated as 1/2 LOD.

e) NA = not analyzed.

f) Data from Bidleman et al., 2014.

**Table S6. Comparison of mean pesticide concentrations in air at Holmön-Krycklan (H-K) and monitoring stations, pg $m^{-3}$.**

| Compound[a] | 2011-12 H-K Sweden | 2010-11 Råö Sweden | 2010-11 Aspvreten Sweden | 2010-11 Pallas[b] Finland | 2005 Alert[b] Canada | 2006 Zeppelin[b] Norway |
|---|---|---|---|---|---|---|
| γ-HCH | 4.5 | 3.1 | 1.8 | 1.5 | 1.7 | 1.9 |
| α-HCH | 7.3 | 4.1 | 4.0 | 5.0 | 13 | 11 |
| ENDO-I | 2.2 | 3.2 | NR | 11.4 | 5.6 | NR |
| ENDO-II | 0.033 | 0.084 | NR | 0.062 | NR | NR |
| ENSUL | 0.063 | 0.079 | NR | 0.31 | NR | NR |
| DIEL | 1.7 | NR[c] | NR | NR | 0.81 | NR |
| DDE | 1.1 | 2.6 | 2.0 | 0.48 | 0.30 | 1.2 |
| HCB | 44 | NR | NR | NR | 52 | 72 |
| TC | 0.47 | 0.18 | 0.25 | 0.14 | 0.25 | 0.22 |
| CC | 0.63 | 0.41 | 0.23 | 0.39 | 0.64 | 0.61 |
| TN | 0.51 | 0.45 | 0.30 | 0.34 | 0.35 | 0.60 |
| HEPX | 0.90 | NR | NR | NR | 0.84 | NR |

a) Abbreviations in Table S1.
b) Pallas, Alert and Zeppelin are air monitoring stations of the Arctic Monitoring and Assessment Program (Hung et al., 2010).
c) NR; not reported.

**Table S7. Temperatures for flux estimates and gas exchange loadings to Bothnian Bay[a,b]**

| | Temperature, K | | | | | Gas exchange loadings, kg[b] | | | | | |
|---|---|---|---|---|---|---|---|---|---|---|---|
| | air | | water | | | Present | Present | Present | Future | Future | Future |
| | Present | Future | Present | Future | Compound[c] | Volatilisation | Deposition | Net | Volatilization | Deposition | Net |
| **January** | 267 | 273 | 273 | 275 | **2,4,6-TBA[d]** | -1090 | 26 | -1060 | NA[e] | NA | NA |
| **February** | 264 | 270 | 273 | 275 | **2,4-DBA[d]** | -214 | 36 | -178 | NA | NA | NA |
| **March** | 272 | 277 | 273 | 275 | **2,6-DBA[d]** | -57 | NA | NA | NA | NA | NA |
| **April** | 275 | 279 | 273 | 275 | **HCB** | -53 | 51 | -2.2 | -80 | 78 | -1.8 |
| **May** | 280 | 284 | 278 | 281 | **ΣHCHs** | -45 | 48 | 3.2 | -71 | 72 | 1.8 |
| **June** | 285 | 288 | 281 | 285 | **ΣCHLs** | -17 | 4.8 | -12 | -26 | 7.2 | -19 |
| **July** | 289 | 292 | 288 | 292 | **HEPX** | NA | 3.5 | NA | NA | 10 | NA |
| **August** | 289 | 292 | 290 | 294 | **DDE** | NA | 4.0 | NA | NA | 4.8 | NA |
| **September** | 285 | 289 | 286 | 289 | **DIEL** | -6.9 | 6.7 | -0.2 | -11 | 5.6 | -0.5 |
| **October** | 279 | 283 | 281 | 284 | **ΣENDOs** | -2.4 | 9.2 | 6.8 | -3.7 | 14 | 10 |
| **November** | 277 | 282 | 278 | 281 | **CPF** | NA | 0.29 | NA | NA | 0.44 | NA |
| **December** | 271 | 277 | 276 | 278 | **DAC** | -0.39 | 0.40 | 0.010 | -0.60 | 0.60 | 0.00 |

a) Area 38000 $km^2$.

b) Annual for pesticides, May-September only for BAs.

c) Group abbreviations: ΣHCH = α-HCH + γ-HCHs, ΣCHLs = TC + CC + TN + CN, ΣENDOs = ENDO-I + ENDO-II + ENSUL.
Single compound abbreviations in Table 1.

d) Loadings from Bidleman et al., 2014.

e) NA: not assessed.

**Table S8. Bulk deposition fluxes[a], ng $m^{-2}$ $month^{-1}$**

| Compound[b] | Krycklan[c] Sweden 2009-10 | Abisko[c] Sweden 2009-10 | Råö[d] Sweden 2010-11 | Aspvreten[d] Sweden 2010-11 | Pallas[d] Finland 2010-11 | Arithmetic mean 2010-11 | Geometric mean 2010-11 |
|---|---|---|---|---|---|---|---|
| α-HCH | 1.9 | 21.8 | 2.2 | 1.5 | 1.9 | 5.9 ± 8.9 | 3.0 |
| γ-HCH | 11.8 | 109 | 6.4 | 2.4 | 2.0 | 26 ± 46 | 8.3 |
| TC | 0.25 | 0.73 | 0.17 | 0.098 | 0.095 | 0.27 ± 0.26 | 0.20 |
| CC | 0.31 | 0.62 | 0.14 | 0.11 | 0.10 | 0.26 ± 0.22 | 0.20 |
| TN | 0.32 | 0.23 | 0.16 | 0.14 | 0.10 | 0.19 ± 0.09 | 0.18 |
| HEPX | 0.85 | 0.41 | NR | NR | NR | 0.63 ± 0.31 | 0.59 |
| DDE | NR[e] | NR | 4.9 | 1.4 | 0.78 | 2.4 ± 2.2 | 1.7 |
| ENDO-I | NR | NR | 2.6 | NR | 8.5 | 5.6 ± 4.2 | 4.7 |
| ENDO-II | NR | NR | 2.1 | NR | 1.4 | 1.8 ± 0.5 | 1.7 |
| ENSUL | NR | NR | 4.0 | NR | 7.8 | 5.9 ± 2.7 | 5.6 |

a) Bulk = precipitation + dry particle deposition. Numbers for each station are arithmetic means.
b) Abbreviations in Table 1.
c) Newton et al., 2014.
d) New data presented here, at EMEP stations discussed by Hansson et al. (2006).
e) NR: not reported.

**Table S9. Atmospheric loadings of halogenated compounds to Bothnian Bay and catchment, kg[a]**

| Compound[c] | deposition[b] bulk[d] | Bothnian Bay gas exchange[e] | total | catchment bulk | system total | volatilization[b] Bothnian Bay gas exchange[f] | system net |
|---|---|---|---|---|---|---|---|
| **HCB** | NA[g] | 51 | NA | NA | NA | -53 | NA |
| **ΣHCHs** | 5.2 | 48 | 54 | 38 | 92 | -45 | 46 |
| **ΣCHLs** | 0.26 | 4.8 | 5.1 | 1.9 | 7.0 | -17 | -10 |
| **HEPX** | 0.27 | 3.5 | 3.8 | 2.0 | 5.8 | NA | NA |
| **DDE** | 0.80 | 4.0 | 4.8 | 5.9 | 11 | NA | NA |
| **ΣENDOs** | 5.5 | 9.2 | 15 | 40 | 55 | -2.4 | 53 |
| **DIEL** | NA | 6.7 | NA | NA | NA | -6.9 | NA |
| **CPF** | NA | 0.29 | NA | NA | NA | NA | NA |
| **DAC** | NA | 0.40 | NA | NA | NA | -0.60 | NA |
| **ΣBAs[h]** | NA | 62 | NA | NA | NA | -1360 | NA |

a) Bothnian Bay: 38000 $km^2$, catchment 280 000 $km^2$, system 318 000 $km^2$.
b) Annual for pesticides, May-September only for BAs (Bidleman et al., 2014).
c) Abbreviations in Tables S1 and S7.
d) Precipitation + particle dry deposition, Table S8.
e) Deposition component of air-sea gas exchange, Table S7.
f) Volatilization component of air-sea gas exchange, Table S7.
g) This pathway was not assessed.
h) 2,4-DBA + 2,6-DBA + 2,4,6-TBA.

**References**


Bidleman, T.F., K. Agosta, A. Andersson, P. Haglund, O. Nygren, M. Ripszam and M. Tysklind, M. 2014. Air-water exchange of brominated anisoles in the northern Baltic Sea. *Environmental Science and Technology* 48, 6124-6132.

Bohlin, P., O. Audy, L. Škrdlíková, P. Kukučka, P. Přibylová, R. Prokeš, Š. Vojta and J. Klánová. 2014. Outdoor passive air monitoring of semi-volatile organic compounds (SVOCs): a critical evaluation of performance and limitations of polyurethane foam (PUF) disks. *Environmental Science Processes and Impacts* 16: 433-444. doi: 10.1039/c3em00644a.

Cetin, B., S. Ozer, A. Sofuoglu and M. Odabasi. 2006. Determination of Henry's law constants of organochlorine pesticides in deionized and saline water as a function of temperature. *Atmospheric Environment* 40: 4538-4546. doi:10.1016/j.atmosenv.2006.04.009.

Gouin, T., T. Harner, P. Blanchard and D. Mackay. 2005. Passive and active air samplers as complementary methods for investigating persistent organic pollutants in the Great Lakes Basin. *Environmental Science and Technology* 39: 9115-9122. doi: 10.1021/es051397f.

Hansson, K., A. Palm Cousins, E. Brorström-Lundén and S. Leppanen. 2006. *Atmospheric concentrations in air and deposition fluxes of POPs at Råö and Pallas, trends and seasonal and spatial variations*, Swedish Environmental Research Institute, Gothenburg, Sweden.

Hung, H., R., Kallenborn, K. Breivik, Y. Su, E. Brorström-Lundén, K. Olafsdottir, J.M.Thorlacius, S. Leppänen, R. Bossi, H. Skov, S. Manø, G.W. Patton, G. Stern, E. Sverko and P. Fellin. 2010.. Atmospheric monitoring of organic pollutants in the Arctic under the Arctic Monitoring and Assessment Programme (AMAP): 1993–2006. *Science of the Total Environment* 408: 2854-2873, doi:10.1016/j.scitotenv.2009.10.044.

Jantunen, L.M.M. and T.F. Bidleman. 1998. Organochlorine pesticides and enantiomers of chiral pesticides in Arctic Ocean water. *Archives of Environmental Contamination and Toxicology* 35: 218-228.

Jantunen, L.M. and T.F. Bidleman. 2006. Henry's law constants for hexachlorobenzene, *p,p'*-DDE and components of technical chlordane and estimates of gas exchange for Lake Ontario. *Chemosphere* 62: 1689-1696. doi:10.1016/j.chemosphere.2005.06.035.

Jantunen, L.M., H. Kylin and T.F. Bidleman. 2004. Air–water gas exchange of α- hexachlorocyclohexane enantiomers in the South Atlantic Ocean and Antarctica. *Deep Sea Research II* 51: 2661-2672. doi:10.1016/j.dsr2.2004.02.002.

Mackay, D. and A.T.K. Yeun. 1983. Mass transfer coefficients for volatilization of organic solutes from water. *Environmental Science and Technology* 17: 211-217.

Muir, D.C.G., C. Teixeira and F. Wania. 2004. Empirical and modeling evidence of regional atmospheric transport of current-use pesticides. *Environmental Toxicology and Chemistry* 23: 2421–2432.

Newton, S., T.F. Bidleman, M. Bergknut, J. Racine,, H. Laudon, R. Giesler and K. Wiberg. 2014. Atmospheric deposition of persistent organic pollutants and chemicals of emerging concern at two sites in northern Sweden. *Environmental Science Processes and Impacts* 16: 298-305. doi: 10.1039/c3em00590a.

Ripszam, M. and P. Haglund. 2015. Automated method for determination of dissolved organic carbon − water distribution constants of structurally diverse pollutants using pre-equilibrium solid phase microextraction. *Environmental Toxicology and Chemistry* 34:, 266-274. doi: 10.1002/etc.2805.

Sahsuvar, L., P.A. Helm, L.M. Jantunen and T.F. Bidleman. 2003. Henry's law constants for α-, β-, and γ-hexachlorocyclohexanes (HCHs) as a function of temperature, and revised estimates of gas exchange in arctic regions. *Atmospheric Environment 37*: 983-992, doi:10.1016/S1352-2310(02)00936-6.

Shen, L. and F. Wania. 2005. Compilation, evaluation, and selection of physical-chemical property data for organochlorine pesticides. *Journal of Chemical and Engineering Data* 50: 742-768, doi: 10.1021/je049693f.

Shoeib, M. and T. Harner. 2002. Characterization and comparison of three passive air samplers for persistent organic pollutants. *Environmental Science and Technology* 36: 4142-4151. doi: 10.1021/es020635t.

Wanninkhof, R. and W.R. McGillis. 1999. A cubic relationship between air-sea $CO_2$ exchange and wind speed. *Geophysical Research Letters* 26: 1889-1892.

Weber, J., C.J. Halsall, D. Muir, C. Teixeira, J. Small, K. Solomon, M. Hermanson, H. Hung and T. Bidleman. 2010. Endosulfan, a global pesticide: A review of its fate in the environment and occurrence in the Arctic. *Science of the Total Environ*ment 408: 2966-2984. doi: 10.1016/j.scitotenv.2009.10.077.

Xiao, H., N. Li and F. Wania. 2004. Compilation, evaluation, and selection of physical-chemical property data for α-, β-, and γ-hexachlorocyclohexane. *Journal of Chemical and Engineering Data* 49: 173-185, doi: 10.1021/je034214i.
